# A systematic review and meta-analysis on the DSM-5 severity specifiers for eating disorders

**DOI:** 10.1192/j.eurpsy.2021.1865

**Published:** 2021-08-13

**Authors:** A. Dang, S. Giles, F. Fernandez-Aranda, L. Kiropoulos, M. Fuller-Tyszkiewicz

**Affiliations:** 1 Melbourne School Of Psychological Science, University of Melbourne, Melbourne, Australia; 2 Ciber Fisiopatología Obesidad Y Nutrición (ciberobn), Instituto Salud Carlos III, Barcelona, Spain; 3 Department Of Psychiatry, University Hospital of Bellvitge-IDIBELL, Barcelona, Spain; 4 School Of Psychology, Deakin University, Melbourne, Australia; 5 Centre For Social And Early Emotional Development, Deakin University, Burwood, Australia

**Keywords:** anorexia nervosa, DSM-5 severity indicators, eating disorders, binge eating disorder

## Abstract

**Introduction:**

The DSM-5 introduced severity indicies for the first time.

**Objectives:**

We conducted a systematic review and synthesis the frequency of each DSM-5 severity categories (i.e., mild, moderate, severe and extreme severe) for Anorexia Nervosa (AN), Bulimia Nervosa (BN) and Binge Eating Disorders (BED), and to evaluate studies that assess the clinical utility of these severity specifiers for all eating disorders (ED) subtypes.

**Methods:**

Five databases (EMBASE, MEDLINE, PsycARTICLES, PsycINFO, and ProQuest) were used to identify for both academic and grey literature published from 2013 until July 8, 2020. Twenty-five studies were retrieved for the systematic review based on the inclusion and exclusion criteria, and up to six studies were qualified for meta-analysis

**Results:**

We found limited support for the current DSM-5 severity ratings for all ED indices, as the majority of ED severity groups were not significantly distinguishable in overall ED psychopathology (mean effect size ranged from .02 to .5). The value of the DSM-5 severity ratings was further devalued as 56.91% to 80.52% of individuals with AN, BN, and BED were categorized into mild and moderate groups. However, there was significant heterogeneity between the studies (p< .001), and some of these heterogeneities were explained by differences in study settings and measurement of eating disorder psychopathology.

**Conclusions:**

Overall, the current study provided little support for the DSM-5 severity ratings for EDs, thus it is suggested that further exploration in alternative severity classification approach is needed.

**Disclosure:**

No significant relationships.

